# CDK4/6 inhibition promotes immune infiltration in ovarian cancer and synergizes with PD-1 blockade in a B cell-dependent manner

**DOI:** 10.7150/thno.44871

**Published:** 2020-08-25

**Authors:** Qian-Feng Zhang, Jia Li, Kuo Jiang, Rui Wang, Jun-li Ge, Hong Yang, Shu-Juan Liu, Lin-Tao Jia, Lei Wang, Bi-Liang Chen

**Affiliations:** 1 Department of Obstetrics and Gynecology, Xijing Hospital, The Fourth Military Medical University, Xi'an, China;; 2 Department of Spine Surgery, Honghui Hospital, Xi'an Jiaotong University, Xi'an, China;; 3 School of Public Health, College of Medicine, Xi'an Jiaotong University, Xi'an, China;; 4 State Key Laboratory of Cancer Biology, Department of Biochemistry and Molecular Biology, The Fourth Military Medical University, Xi'an, China

**Keywords:** CDK4/6, immune infiltration, chemokine, PD-1, ovarian cancer

## Abstract

Great progress has been made in the field of tumor immunotherapy in the past decade. However, the therapeutic effects of immune checkpoint blockade (ICB) against ovarian cancer are still limited. Recently, an inhibitor of cyclin-dependent kinases 4 and 6 (CDK4/6i) has been reported to enhance antitumor immunity in preclinical models. The combined use of CDK4/6i and ICB may be beneficial, but the effects of CDK4/6is on the tumor immune microenvironment and whether they can synergize with ICB in treating ovarian cancer remain unknown.

**Methods:** In this study, we first assessed the antitumor efficacy of abemaciclib, an FDA-approved CDK4/6i, in a syngeneic murine ovarian cancer model. Then, immunohistochemistry, immunofluorescence and flow cytometry were performed to evaluate the number, proportion, and activity of tumor-infiltrating lymphocytes. Cytokine and chemokine production was detected both *in vivo* and *in vitro* by PCR array analysis and cytokine antibody arrays. The treatment efficacy of combined abemaciclib and anti-PD-1 therapy was evaluated *in vivo*, and CD8+ and CD4+ T cell activities were analyzed using flow cytometry. Lastly, the requirement for both CD8+ T cells and B cells in combination treatment was evaluated *in vivo,* and potential cellular mechanisms were further analyzed by flow cytometry.

**Results:** We observed that abemaciclib monotherapy could enhance immune infiltration, especially CD8+ T cell and B cell infiltration, in the ID8 murine ovarian cancer model. Immunophenotyping analysis showed that abemaciclib induced a proinflammatory immune response in the tumor microenvironment. PCR array analysis suggested the presence of a Th1-polarized cytokine profile in abemaciclib-treated ID8 tumors. *In vitro* studies showed that abemaciclib-treated ID8 cells secreted more CXCL10 and CXCL13, thus recruiting more lymphocytes than control groups. Combination treatment achieved better tumor control than monotherapy, and the activities of CD8+ and CD4+ T cells were further enhanced when compared with monotherapy. The synergistic antitumor effects of combined abemaciclib and anti-PD-1 therapy depended on both CD8+ T cells and B cells.

**Conclusion:** These findings suggest that combined treatment with CDK4/6i and anti-PD-1 antibody could improve the efficacy of anti-PD-1 therapy and hold great promise for the treatment of poorly immune-infiltrated ovarian cancer.

## Introduction

Ovarian cancer is the most lethal gynecological malignancy in the world. Owing to its occult onset and rapid development, approximately 70% of ovarian cancer cases are diagnosed at an advanced stage. For those patients, the five-year survival rate is less than 30% 1. Currently, the first-line treatment for ovarian cancer is still debulking surgery followed by platinum-based chemotherapy. Although new therapies, such as antiangiogenic agents and Poly(ADP-ribose) polymerase inhibitors, have been approved by the FDA, the overall survival of ovarian cancer patients remains largely unchanged 2.

Immune checkpoint blockade therapies represented by PD-1/PD-L1 inhibition have shown great promise in treating various kinds of solid tumors, such as melanoma and non-small cell lung cancer 3. However, their therapeutic effects against ovarian cancer are still limited. According to reported clinical trials, the overall response rate is only 15% 4. Potential mechanisms underlying the limited efficacy of immune checkpoint inhibitors in ovarian cancer involve many aspects, among which limited lymphocyte infiltration and immunosuppressive tumor microenvironment are nonnegligible factors 5,6. Thus, it is necessary to develop combination treatment strategies to improve the response of ovarian cancer to immunotherapy.

Increasing evidence indicates that small-molecule inhibitors targeting oncogenic pathways can exert effects beyond the biological behavior of tumor cells. Some small-molecule inhibitors can directly modulate the immune microenvironment of tumor tissues and promote immune-mediated tumor destruction 7,8. For example, a BRAF inhibitor, when combined with an anti-PD-1 or anti-PD-L1 antibody, can increase the number and activity of tumor-infiltrating lymphocytes (TILs) in melanoma, leading to improved tumor control and patient survival 9. Due to the dual effects of small-molecule drugs on both tumor cells and the immune microenvironment, the combined use of small-molecule inhibitors and ICB could be a promising strategy for cancer treatment.

Aberrant activation of cyclin-dependent kinases (CDKs) is strongly correlated with tumor malignancy 10, and CDK4/6 inhibitors (CDK4/6is) have already been approved by the FDA for the treatment of breast cancer. Moreover, there is increasing evidence showing the immunomodulatory effects of CDK4/6is *in vivo*, including enhanced antigen presentation, increased infiltration of CD8+ T cells and reduced regulatory T cell (Treg) proliferation, which are beyond tumor cell cycle control 11-13. Interestingly, tumor cell senescence induced by CDK4/6i treatment shows a proinflammatory secretory phenotype, which is associated with increased lymphocyte infiltration 14,15. Thus, CDK4/6is have the potential to enhance the immune-mediated elimination of tumor cells when combined with immunotherapy. Aberrant activation of CDK4 and CDK6 is a common finding in ovarian cancer 16,17. Emerging evidence also shows that CDK4/6is suppress ovarian cancer progression both* in vitro* and* in vivo* 18-20. In ovarian cancer patients, CDK4/6is have achieved stable disease or a durable CA-125 response 21. Based on these studies, CDK4/6 inhibition could be a promising strategy in ovarian cancer. However, the effects of CDK4/6is on the tumor microenvironment (TME) of ovarian cancer and whether these inhibitors can enhance the efficacy of immunotherapy are still unknown.

In this study, we evaluated the antitumor efficacy of an FDA-approved CDK4/6i and its effect on the immune microenvironment in a syngeneic murine ovarian cancer model. Then we explored the underlying cellular and molecular mechanisms. We show that the combination of CDK4/6i and PD-1 blockade has synergistic activity in the treatment of ovarian cancer. The mechanism underlying this synergy was related to the increased infiltration of T cells and B cells caused by CDK4/6 inhibition.

## Materials and Methods

### Mice and Tumor models

Six-week-old female C57BL/6 mice were purchased and housed under standard pathogen-free conditions in the animal center of Fourth Military Medical University. ID8, a cell line widely used to establish a syngeneic mouse model of human ovarian cancer 22, was purchased from Merck Millipore (Cat. No. SCC145) and maintained in high-glucose Dulbecco's modified Eagle's medium (DMEM; Gibco) supplemented with 4% fetal bovine serum (FBS; Gibco) and 5 μg/mL insulin (Gibco).

To establish *in vivo* models, 5×10^6^ luciferase-tagged ID8 (ID8-luc) cells were intraperitoneally injected into Six-week-old C57BL/6 mice. Three weeks later, all mice were divided into the required groups after confirmation of tumor formation with the In Vivo Imaging System (IVIS; Caliper Life Science, Hopkinton, MA). Tumor progression was monitored with the IVIS every week. All animal experiments were approved by the Laboratory Animal Welfare and Ethics Committee of Fourth Military Medical University.

### Inhibitors and antibodies

A selective CDK4/6i, abemaciclib, was purchased from Selleck (Houston, TX, USA). An *in vivo* anti-mouse PD-1 antibody (clone RMP1-14) was purchased from BioXCell (West Lebanon, NH, USA).

### Immunohistochemistry (IHC) and immunofluorescence (IF)

IHC and IF were performed on formalin-fixed, paraffin-embedded tissue samples. The procedure for IHC was described previously 23. The primary antibodies used included rabbit anti-mouse CD45 (1:200, CST, 70257), rabbit anti-mouse CD8α (1:400, CST, 98941), rabbit anti-mouse CD19 (1:800, CST, 90176), and rabbit anti-mouse PD-L1 (1:200, CST, 64988). For IF, sections were stained with rat anti-mouse CD3 (1:100, Abcam, ab56313) and rabbit anti-mouse CD19 (1:800, CST, 90176) antibodies, followed by staining with goat anti-rat (Abcam, ab150165) and goat anti-rabbit (Abcam, ab150088) antibodies. DAPI (Invitrogen) was added to counterstain the nuclei. Finally, images were acquired using a Nikon A1R confocal laser scanning microscope system and analyzed using ImagePro software.

### TIL extraction and flow cytometry

Mice were euthanized on day 10 after treatment initiation, and tumor tissues were harvested, washed in 2 mL of DMEM, finely minced into 2- to 4-mm pieces and digested with the gentleMACS Dissociator (Miltenyi Biotech) in a mixed enzyme buffer prepared from a tumor dissociation kit (Miltenyi Biotech). A single-cell suspension was then obtained by passing the mixture through a 70-μm cell mesh. To further enrich TILs, Ficoll-Paque PREMIUM 1.084 (Thermo Fisher Scientific) was added to the bottom of the single-cell suspension, and the suspension was centrifuged at 1,000 × g for 20 min. After centrifugation, TILs were obtained from the interface between the medium and Ficoll-Paque 24.

For phenotypic and functional analyses, enriched TILs were first stimulated with ionomycin (1 μg/mL) and phorbol 12-myristate 13-acetate (20 ng/mL) with Golgi-Stop (BD Biosciences) in DMEM for 4 hours. The cells were then incubated with fragment crystallizable block and stained with surface marker-specific antibodies including anti-CD45 (BioLegend, clone: 30-F11), anti-CD3 (BioLegend, clone: 17A2), anti-CD4 (BD Horizon, clone: RM4-5), anti-CD8 (BD Pharmingen, clone: 53-6.7), anti-CD107a (BD Pharmingen, clone: 1D4B), anti-CD73 (BD Pharmingen, clone: TY/23), anti-CD19 (BD Pharmingen, clone:1D3), anti-B220 (BioLegend, clone: RA3-6B2), anti-CD69 (BD Pharmingen, clone: H1.2F3), anti-IL-10 (BioLegend, clone: JES5-16E3), anti-CD11c (BioLegend, clone: N418), anti-CD40 (BioLegend, clone: 3/23), anti-CD80 (BioLegend, clone: 16-10A1), anti-CD86 (BioLegend, clone: GL-1), anti-F4/80 (BioLegend, clone:BM8), anti-CD206 (BioLegend, clone: C068C2), anti-MHCII (invitrogen, clone: M5/114.15.2), and anti-Gr-1 (BioLegend, clone: RB6-8C5). For intracellular staining, anti-Foxp3 (BD Horizon, clone: MF23), anti-IFN-γ (BD Pharmingen, clone: XMG1.2), anti-T-bet (BioLegend, clone: 4B10), and a fixation/permeabilization solution kit (BD Bioscience) was used before staining. Fixable Viability Stain 510 (BD Horizon) was also used for live/dead cell discrimination. Data were acquired using a BD FACS Canto II and analyzed using FlowJo (TreeStar).

### RT Profiler PCR array

The Mouse Cytokine and Chemokine PCR Array PAMM-150Z (Qiagen) was applied to evaluate transcriptional level changes in genes encoding major Th1 and Th2 cytokines and chemokines. Tumor tissues were harvested 10 days after treatment initiation and stored in an RNAlater stabilization solution. Following RNA extraction (TRIzol™ Plus RNA Purification Kit, Invitrogen), an RT^2^ first-strand kit (Qiagen) was used for cDNA synthesis.

PCR array analysis was performed in accordance with the manufacturer's protocol using the ABI 7500 Real Time PCR System (LifeTech, Glasgow, UK). Acquired data were analyzed with the PCR Array Data Analysis Web Portal of Qiagen. A relative mRNA expression level change (abemaciclib vs control) of more than threefold higher or lower was considered significant.

### Mouse cytokine and chemokine array

ID8 cells seeded at equal numbers were cultured in complete medium for 24 hours, washed twice with PBS and cultured in FBS-free DMEM plus 10 µmol/L abemaciclib or PBS for 24 hours. Then, the supernatants were collected and processed for mouse cytokine array analysis (AAM-CYT-1000, Ray Biotech) according to the manufacturer's protocols. Membranes were scanned using an LAS-500 imager (Fuji, Japan). Relative cytokine levels were obtained by grayscale analysis using ImageJ (NIH).

### Transwell assay and *in vivo* chemokine neutralization

Mouse spleens were dissociated, and red blood cells were removed with a red blood cell lysis buffer. Then, isolation of CD8+ T cells and B cells was performed using a CD8+ T cell isolation kit and B cell isolation kit (both from Miltenyi Biotec). The isolated cells were then used for an *in vitro* transwell assay.

*In vitro* migration of CD8+ T cells and B cells was evaluated in 24-well plates with a polyethylene terephthalate hanging cell culture insert (5.0 μm; Merck Millipore). The bottom chamber contained 600 μL of supernatants of abemaciclib- or PBS-treated ID8 cells as the chemoattractant. For transwell assays utilizing blocking antibodies, 30 μg/mL of anti-CXCL10 (R&D Systems, 134013) or 40 μg/mL anti-CXCL13 (R&D Systems, AF470) were added to the lower chamber containing supernatants of abemaciclib-treated ID8 cells. Rat IgG (R&D Systems, MAB006) or goat IgG (R&D Systems, AB-108-C) were used as isotype control antibodies. Freshly isolated CD8+ T cells or B cells in 100 μL were seeded in the upper chamber. After a 3-hour incubation at 37 °C in a standard 5% CO_2_ incubator, cells that migrated into the lower chamber were counted with a hemocytometer.

For *in vivo* neutralization of CXCL10, 50 µg of Rat anti-mouse CXCL10 was intraperitoneally injected into tumor bearing mice every 3 days for 3 weeks, Rat IgG was used as isotype control. For CXCL13 neutralization, 0.6 mg of goat anti-mouse CXCL13 or goat IgG was intraperitoneally injected once a week for 3 weeks.

### *In vivo* cell depletion

To induce CD8+ T cell depletion, mice were intraperitoneally injected with 500 mg of anti-mouse CD8α antibody (clone YST-169.4, BioXCell) per mouse 3 days before tumor implantation and every 3 days thereafter. For B cell depletion, a combination of 300 mg of anti-mouse CD19 antibody (clone 1D3, BioXCell) and 300 mg of anti-mouse B220 antibody (clone RA3.3A1/6.1, BioXCell) was intraperitoneally injected 3 weeks before tumor inoculation and every 5 days thereafter 25.

### Statistics

Statistical analysis was performed using GraphPad Prism 7. All data are presented as the Mean ± SD. A two-tailed, unpaired T test was used to compare two groups. For multiple comparisons, one-way ANOVA was used. Survival curves were plotted using the Kaplan-Meier method, and statistical probabilities were generated using the log-rank test. To determine whether abemaciclib and anti-PD-1 worked synergistically on tumor control, repeated measurement bioluminescent data in a 2×2 factorial arrangement were analyzed using R statistical software, version 3.6.0. P values less than 0.05 were considered statistically significant (*, *P* < 0.05; **, *P* < 0.01; ***, *P* < 0.001; and ****, *P* < 0.0001).

## Results

### Abemaciclib monotherapy suppresses tumor progression in an ID8 tumor model

**Figure 1:**
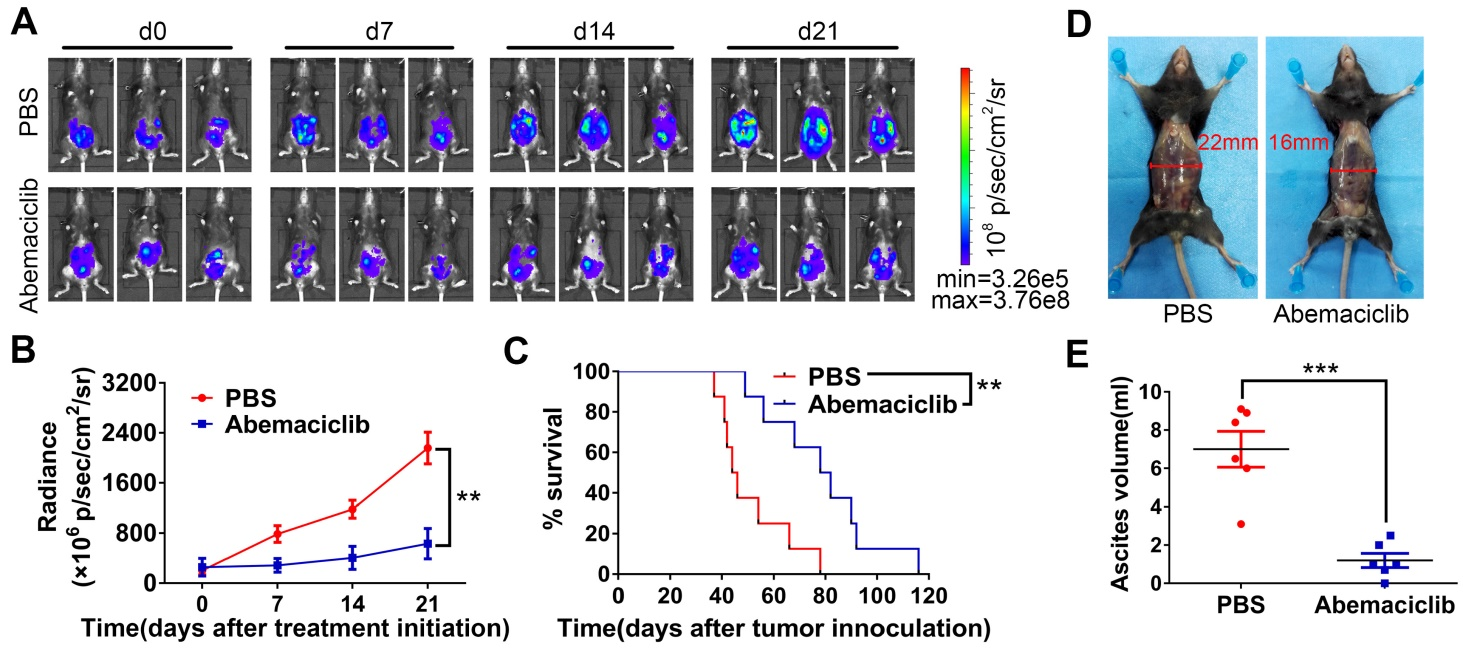
** Abemaciclib controls tumor progression in the ID8 model. (A)** Bioluminescence of C57 mice after inoculation of luciferase-tagged ID8 cells is shown. Treatment began after tumor formation was confirmed, and tumor progression was monitored every 7 days during treatment. Representative images of 3 mice in each group are shown, n=8 mice. d0: day 0, the day before the first treatment. **(B)** The tumor burden was evaluated by quantification of total flux with Living Image software. Data presented are the group mean of 8 mice ± standard error.** (C)** Kaplan-Meier curves show the survival time of the C57 mice included in Figure 1B.** (D)** For the mechanistic study, mice were sacrificed on day 10 post first treatment, and representative pictures show ascites formation in each group. **(E)** Ascites were collected for volume measurement, n=6 mice.

To evaluate the potential and possible mechanism of a CDK4/6i and checkpoint blockade combination for ovarian cancer treatment, a widely used ID8 tumor-bearing mouse model 26,27 was used in our research. A total of 5×10^6^ ID8-luc cells were intraperitoneally injected into immunocompetent syngeneic C57BL/6 mice. Twenty-one days after cell injection, tumor formation was confirmed with an IVIS. Mice were treated with abemaciclib (50 mg/kg) or PBS once daily for a total period of 21 days. Tumor growth was monitored by the IVIS every 7 days (**Figure 1A**). For TME analysis, mice were sacrificed on day 10 post treatment initiation, and tumors were harvested for immunostaining or flow cytometry analysis (n=6). The remaining mice were continually treated with abemaciclib or PBS and followed to monitor long-term survival. Tumor growth was significantly inhibited in the CDK4/6i group compared with the control group (**Figure 1B**). Although tumors began to progress in the late stage of treatment, survival analysis showed that abemaciclib improved the survival of ID8 tumor-bearing mice (**Figure 1C**). Ascites volume measurement on day 10 post treatment initiation also indicated tumor control in the CDK4/6i group (**Figure 1D-E**). These results show that abemaciclib can suppress tumor growth in the ID8 model.

### Abemaciclib monotherapy enhances lymphocyte infiltration in ID8 ovarian tumors

**Figure 2:**
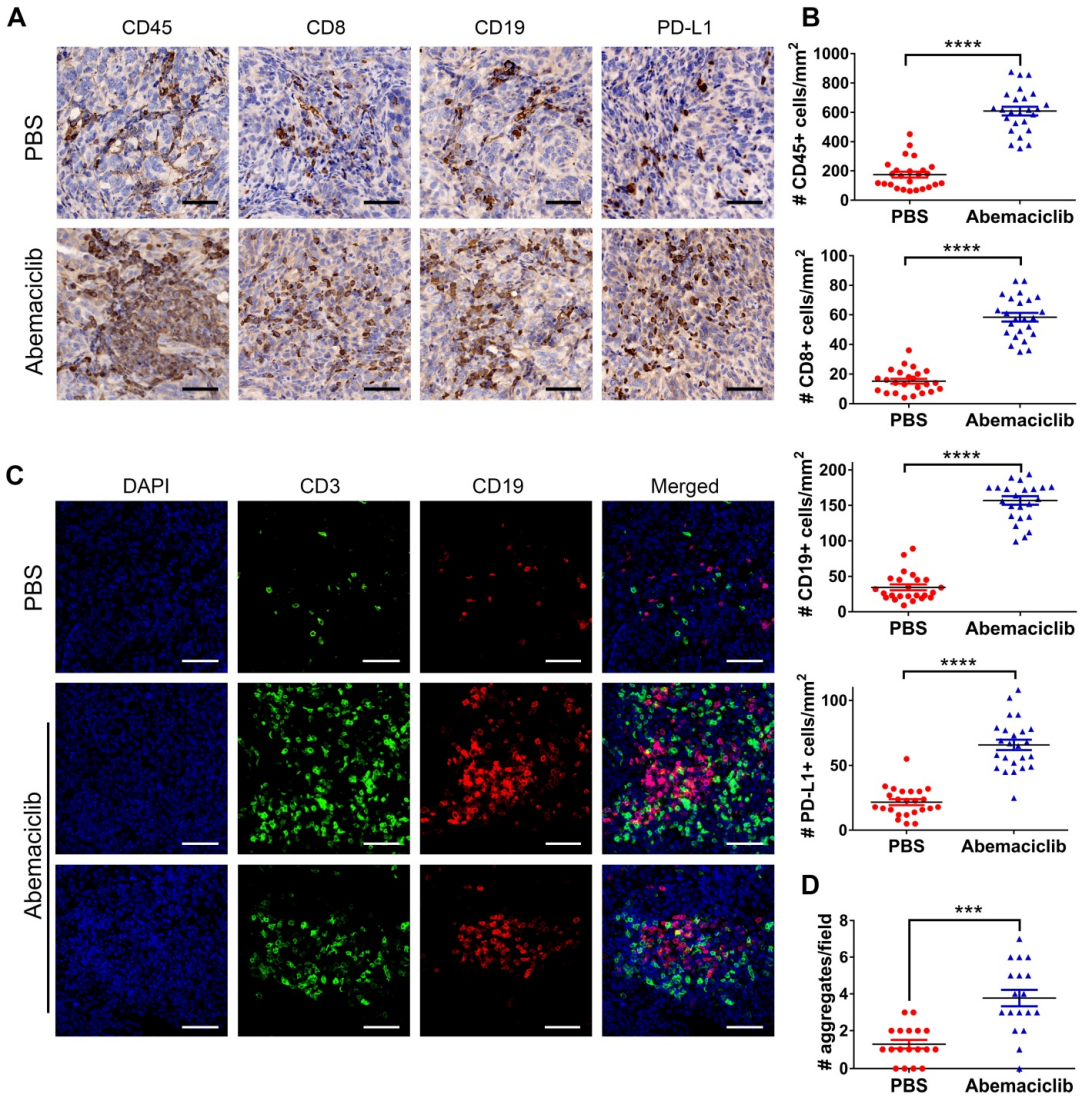
** Abemaciclib treatment induces immune infiltration and T/B cell aggregation. (A)** Representative immunohistochemical staining for CD45, CD8, CD19 and PD-L1 (400×), scale bars: 50 μm. **(B)** TIL was quantified by counting positive signals in four randomly selected fields (400×) in each tumor section using ImageJ, n=6 mice. Statistical comparisons were performed using an unpaired T test. **(C)** Representative immunofluorescence images of CD3 (green), CD19 (red) and nuclear staining (DAPI, blue) showing different types of cell aggregates (400×). Both CD3+ T cells and CD19+ B cells in the control group were scattered, while the numerous infiltrated T cells and B cells formed ectopic lymphoid-like structures with different shapes in the abemaciclib-treated group, scale bars: 50 μm. **(D)** Quantification of T cell/B cell aggregates in 3 randomly selected 100× fields in each tumor section.

To investigate the influence of the CDK4/6i on the ID8 TME, we first examined the expression of CD45, CD8, CD19 and PD-L1 in ID8 tumors by IHC. The results showed that the positive expression of these markers was significantly upregulated in the abemaciclib-treated group compared with the control group (**Figure 2A-B**). Given the TIL aggregation pattern observed by immunohistochemical staining, we further performed IF to assess the distribution patterns of CD3+ T cells and CD19+ B cells in both groups. The results showed that most of the infiltrated T cells and B cells in the PBS groups were scattered, while in the abemaciclib-treated group, clusters of T cell and B cell aggregates were in close contact and formed ectopic lymphoid-like structures (ELS). These structures contained a T cell zone and B cell zone in close contact with each other (**Figure 2C**). The number of T-B cell aggregates in the control group was obviously less than that in the abemaciclib-treated group (**Figure 2D**). Taken together, these results indicate that abemaciclib can significantly increase the infiltration of immune cells into ID8 tumors and promote T-B cell aggregation.

### Abemaciclib treatment induces a proinflammatory immune response in the ID8 model

**Figure 3:**
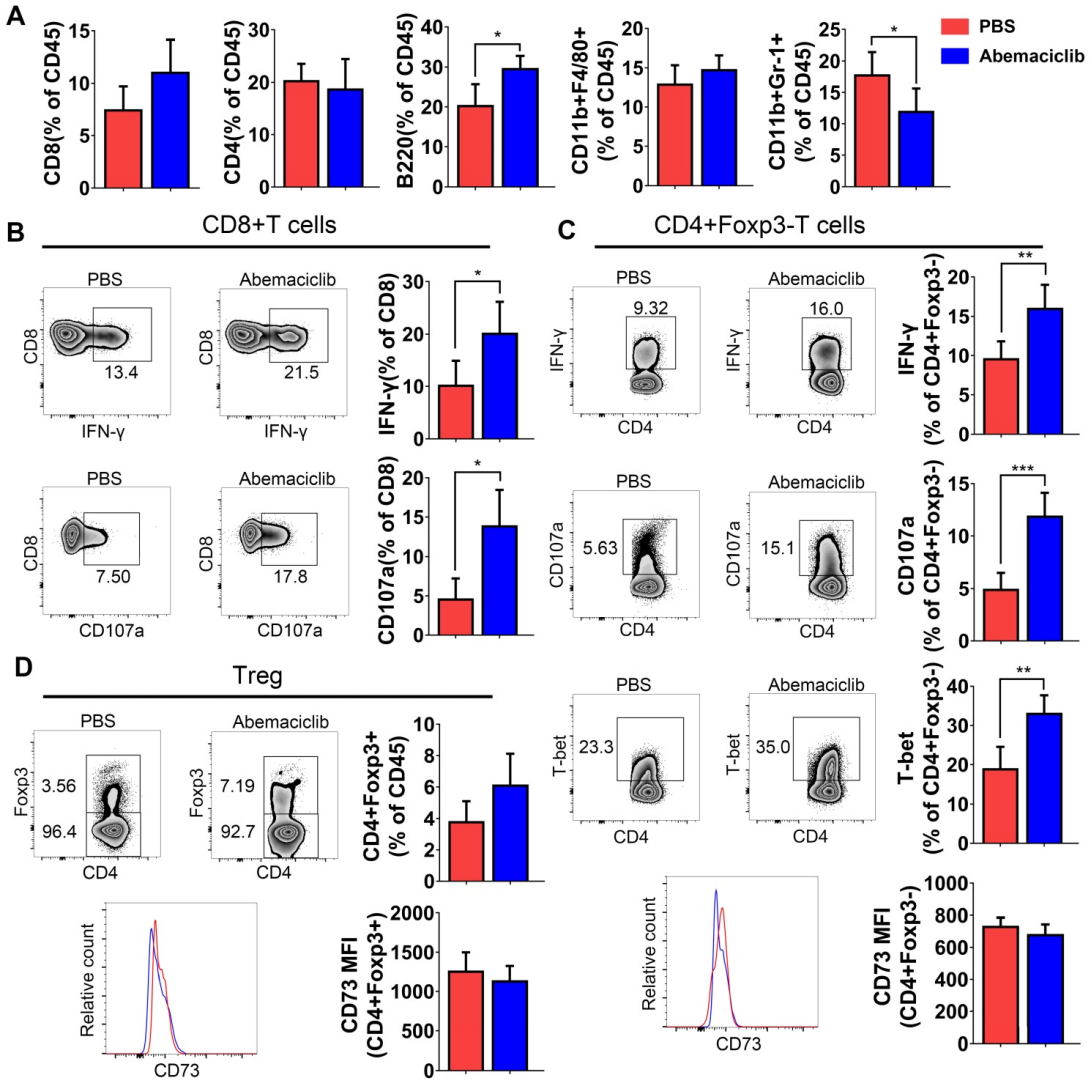
** Proportion of TILs from PBS- or abemaciclib-treated ID8 tumors and phenotypic analysis of T cells by flow cytometry, n=6 mice. (A)** The proportions of CD8+ T cells, CD4+ T cells, B220+ B cells, CD11b+F4/80+ tumor-associated macrophages (TAMs) and CD11b+Gr-1+ MDSCs in the CD45+ TIL population were determined by flow cytometry. **(B)** Representative zebra plots of IFN-γ and CD107a expression in CD8+T cell subsets. **(C-D)** Representative zebra plots of CD4+foxp3- Tregs and CD4+foxp3+ T cells, histogram overlays show CD73 expression in each subset.

Although immunohistochemical analysis showed increases in CD45+, CD8+ and CD19+ cell numbers upon abemaciclib treatment, the proportions of different TIL subpopulations were not obviously changed between the two groups, except for an increase in B cell proportion and a decrease in the CD11B+Gr-1+ myeloid-derived suppressor cell (MDSC) proportion (**Figure 3A**), which indicate that abemaciclib treatment may induce mixed lymphocyte infiltration.

The positive rate of the expression of effector markers (IFN-γ and CD107a) was obviously increased in CD8+ and CD4+foxp3- T cell subsets (**Figure 3B-C**), and the expression of T-bet, an important transcription factor for Th1-type differentiation in CD4+foxp3- T cell subsets, showed an obvious increase (**Figure 3C**). The percentage of CD4+Foxp3+ Tregs showed an increasing trend; however, the increase was not statistically significant. CD73, which indicates the immunosuppressive function of Tregs, did not show a difference in both non-Treg and Treg (**Figure 3C-D**).

**Figure 4:**
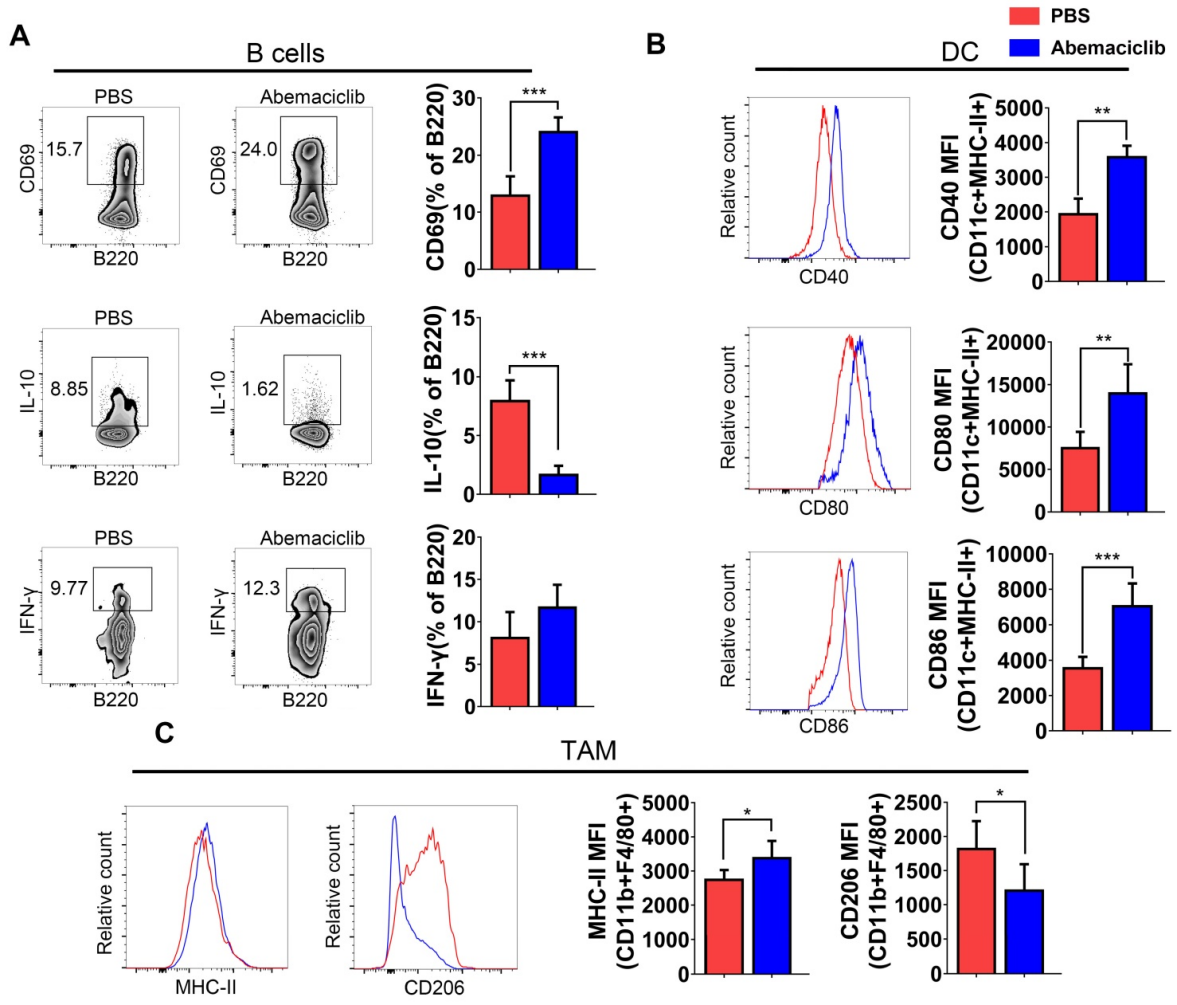
** Phenotypic analysis of B cells, DCs, and TAMs from PBS- or abemaciclib-treated ID8 tumors by flow cytometry, n=6 mice. (A)** Representative zebra plots of activation marker expression and cytokine levels in CD45+CD3-CD11c- B220+ B cells. **(B)** Histogram overlays show different costimulatory molecules expression on CD45+CD11c+MHC-II+ dendritic cells.** (C)** Representative histogram overlay of MHC-II shows M1-polarized TAMs and that of CD206 shows M2-polarized TAMs.

B cell subsets showed not only an increase in their proportion in the TIL population but also the expression of a marker of early activation, CD69. IL-10, an immunosuppressive cytokine, exhibited a decreased level in B cells from inhibitor-treated group. The frequency of IFN-γ producing B cells increased slightly, though not statistically significant (**Figure 4A**). Mature dendritic cells in abemaciclib treated tumors displayed significantly higher expression of co-stimulation markers, CD40, CD80 and CD86 (**Figure 4B**), suggesting enhanced antigen presenting ability of these mature DCs upon CDK4/6 inhibition. CD11b+F4/80+ macrophages play different roles when polarizing to different states. Here, we analyzed the expression of MHCII and CD206 in this subset, and the results indicated that the proportion of MHCII+ cells was increased while that of CD206+ cells was decreased (**Figure 4C**).

### Abemaciclib treatment drives CD8+ T cell and B cell recruitment to the TME

**Figure 5:**
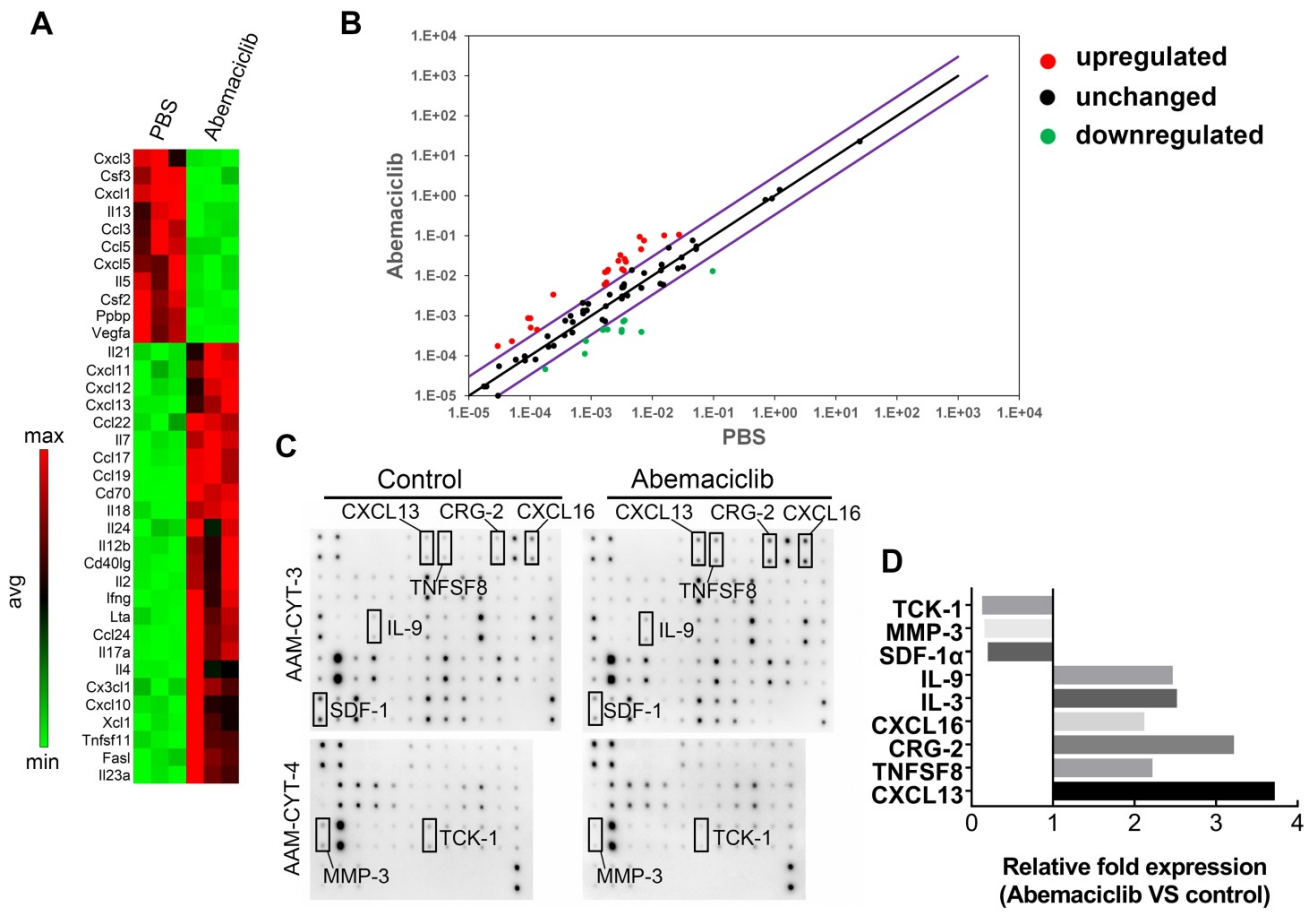
** Abemaciclib treatment changes the chemokine profile in ID8 tumors. (A)** RT^2^ Profiler PCR arrays were used to screen upregulated and downregulated genes after abemaciclib treatment, and the heatmap shows genes with a fold change>3 and *P< 0.05*. **(B)** Scatter plots show the fold changes in gene expression after abemaciclib treatment. The black line indicates fold changes ((2 ^ (-Ct)) of 1, and the violet line indicates fold changes of 3. **(C)** A mouse cytokine array was used to detect chemokine variations in the supernatants of ID8-luc cells after PBS or abemaciclib treatment. n=3. **(D)** Grayscale analysis was used for relative quantification, and chemokines with a fold change>2 and *P< 0.05* are shown.

To investigate the possible reasons for enhanced immune infiltration upon abemaciclib treatment, we used the RT^2^ profiler PCR Array PAMM-150Z to assess immune-related cytokine and chemokine expression at the mRNA level. The results revealed an elevated Th1-type cytokine profile in the abemaciclib-treated group, and the levels of transcripts related to T cell function and recruitment (*Il2*, *Ifng* and *Cxcl10*) were largely upregulated in the abemaciclib-treated group compared with the control group, with fold changes of 7.91, 11.0 and 4.04, respectively. In addition, *Cxcl13* and *Cd40lg*, which function in B cell homing to follicles and T-B cell crosstalk, also exhibited obviously increased expression. With regard to downregulated genes, immunosuppressive cytokine genes* Ppbp* and *Vegfa*, displayed 6.7- and 15.82-fold downregulation, respectively (**Figure 5A**). The results are also shown in scatter plots (**Figure 5B**).

CDK4/6is can induce a “senescence-associated secretory phenotype” 14, which may influence immune infiltration. To investigate whether the increased TIL infiltration and altered cytokine mRNA expression phenotype observed upon abemaciclib treatment are associated with an ID8 cell secretory phenotype, we treated ID8 cells with 10 µmol/L abemaciclib or PBS *in vitro*, and the supernatants were collected for protein detection using the mouse cytokine array C1000 (**Figure 5C**). After grayscale analysis, relative fold expression (abemaciclib vs control) was calculated based on the grayscale value of each point. The results showed 6 upregulated cytokines (IL9, IL3, CXCL16, CRG-2, TNFSF8, and CXCL13) and 3 downregulated cytokines (TCK-1, MMP3, and SDF1-α) (**Figure 5D**). Among these cytokines, TCK-1 (PPBP), CRG-2 (CXCL10) and CXCL13 showed trends that matched those detected by the PCR array.

**Figure 6:**
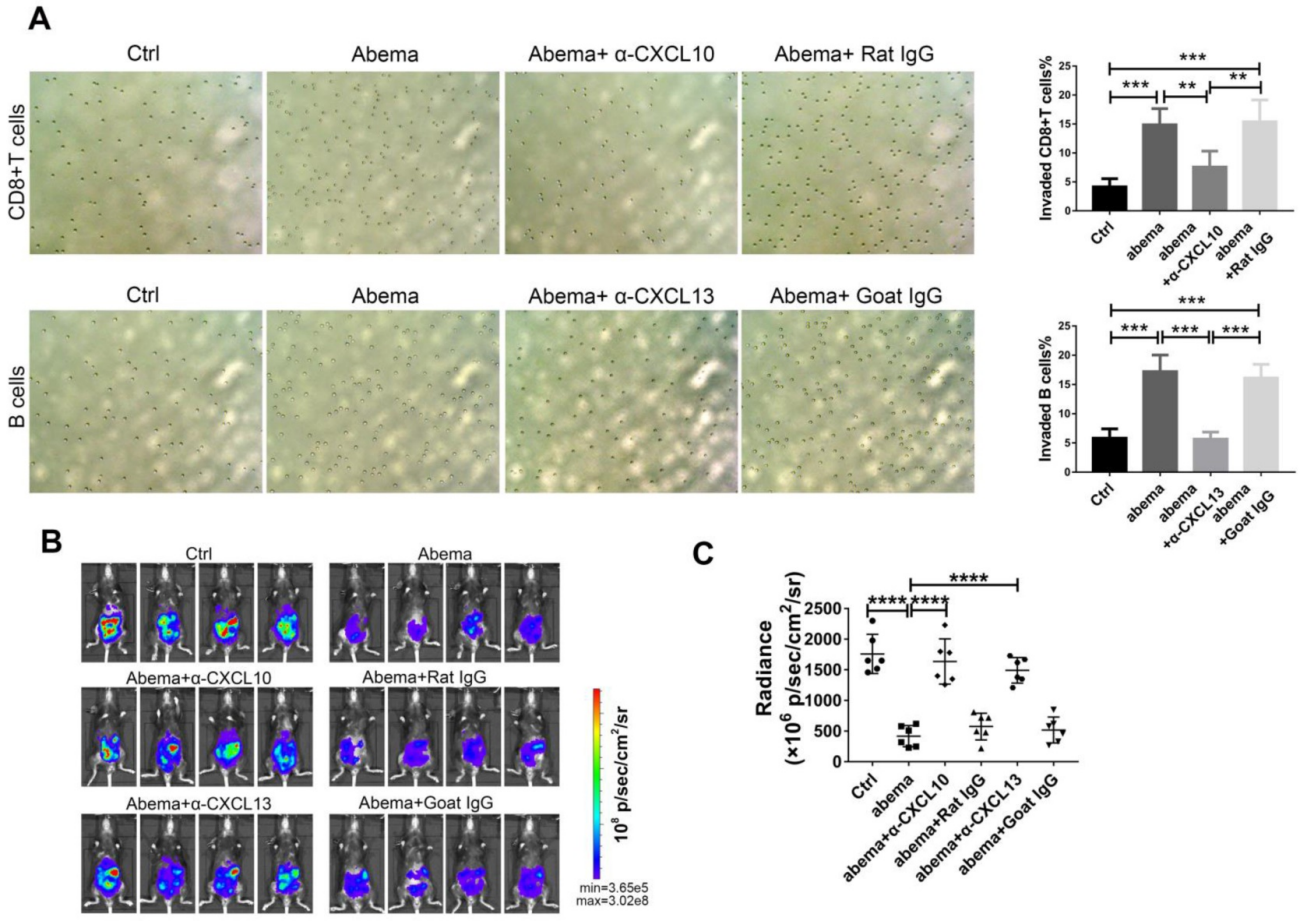
** CXCL10 or CXCL13 neutralization blocked CD8+T cell or B cell migration *in vitro* and abrogated the therapeutic effect of abemacilib *in vivo.* (A)** Transwell assays were performed to detect the chemotactic effect of ID8 cell supernatants after PBS or abemaciclib treatment, and serum-free DMEM was used as the culture medium. Transwell assays were also tested in the presence of neutralizing anti-CXCL10 or anti-CXCL13 antibody. Each sample was analyzed in triplicate. **(B)** Representative images of ID8-luc models at day 21 post first treatment are shown. **(C)** The data were quantified with an IVIS, n=6 mice.

To further investigate whether abemaciclib treatment enhances the chemotactic effect of ID8 supernatant on immune cells, we performed a transwell migration assay using supernatants from abemaciclib- or PBS-treated ID8 cells cultured in FBS-free DMEM medium as the chemoattractant. The upper chambers were seeded with freshly isolated mouse splenic CD8+ T cells or B cells. The results showed that the percentage of migrated lymphocytes in the abemaciclib-treated group was higher than that in the control group. In addition, adding an anti-CXCL10 or anti-CXCL13 antibody into the transwell system largely reduced the chemotaxis caused by abemaciclib treatment (**Figure 6A**). Furthermore, *in vivo* neutralization of CXCL10 or CXCL13 dampened the therapeutic effect of abemaciclib (**Figure 6B-C**), indicating that CXCL10-mediated CD8+T cell infiltration and CXCL13-mediated B cell infiltration paly crucial roles in abemaciclib induced tumor suppression. Taken together, these results suggest that abemaciclib-treated ID8 tumors may enhance chemotaxis of CD8+T cells and B cells via increased chemokine secretion.

### Abemaciclib synergizes with anti-PD-1 therapy to achieve better tumor control in the ID8 model

**Figure 7:**
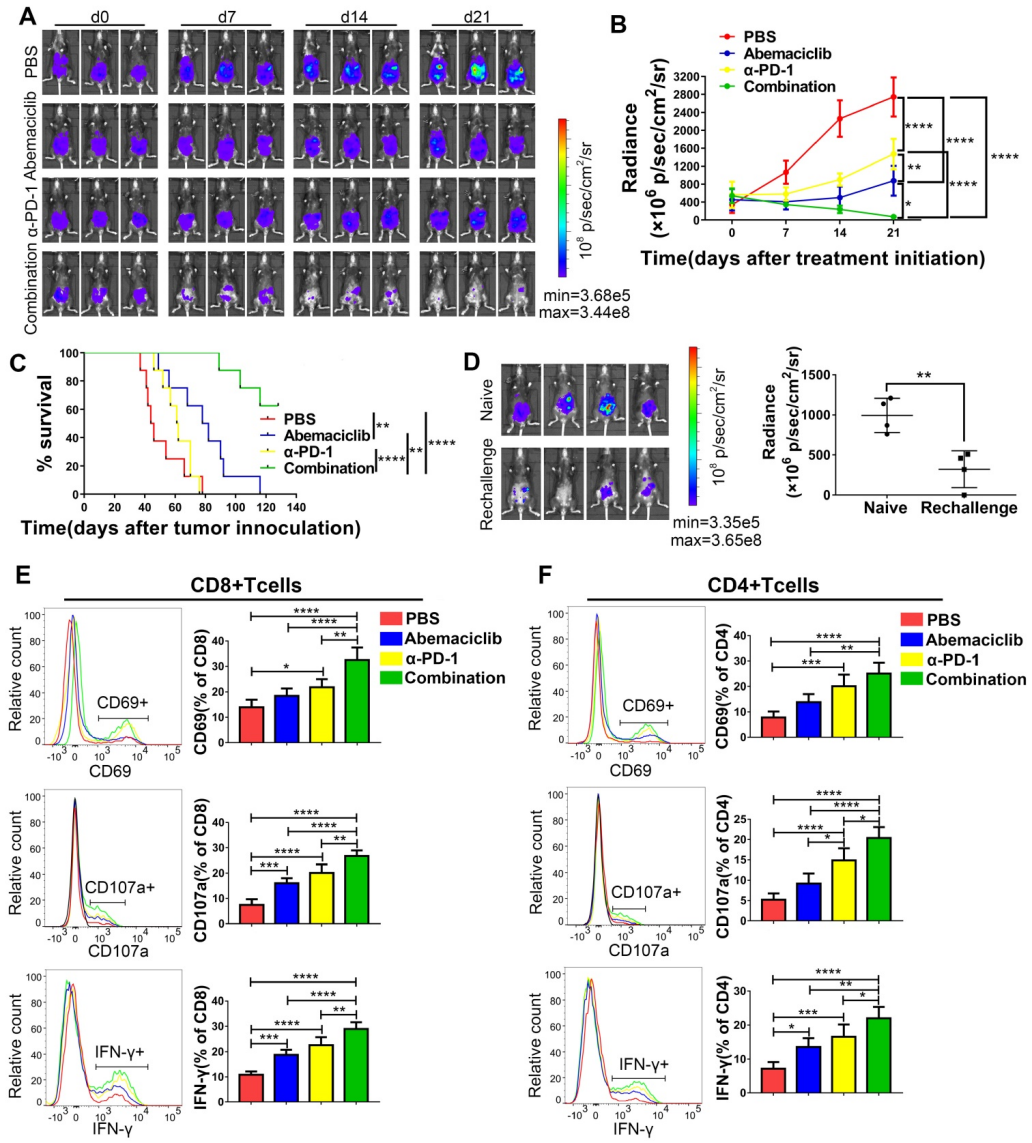
** Abemaciclib synergizes with anti-PD-1 therapy in ID8 tumor control, and combination therapy achieves longer survival than monotherapy. (A)** Tumor progression was monitored every 7 days during treatment, and representative images of 3 mice in each group are shown; n=8 mice. **(B)** The tumor burden was evaluated by quantification of total flux with Living Image software. Data presented are the group mean of 8 mice ± standard error. **(C)** The long-term survival of ID8 tumor-bearing mice treated with PBS, abemaciclib or an anti-PD-1 mAb alone or in combination is shown. **(D)** Bioluminescence images of mice on day 14 after tumor cell re-challenge, naïve C57 mice were used as controls(left). The tumor burden was evaluated by quantification of total flux (right). **(E-F)** Flow cytometry analysis of functional and activation markers expressed by CD8+ T cells (left) or CD4+ T cells (right) in ID8 tumors at day 10 post treatment initiation, n=6 mice. Error bars, SD.

Based on the enhanced CD8+ T cell infiltration we observed, which suggests the potential for combining abemaciclib with immunotherapy that unleashes T cell activity, we assessed the combinatorial efficacy of abemaciclib and an anti-PD-1 mAb. Three weeks after cell implantation, mice were peritoneally injected with abemaciclib (50 mg/kg in 200 µL), an anti-PD-1 mAb (200 µg in 100 µL per injection), abemaciclib plus the anti-PD-1 mAb, or PBS (200 µL per injection). Abemaciclib was administered once daily and the anti-PD-1 mAb was administered every 3 days for a total period of 21 days for long-term survival studies and 10 days for a mechanistic study. Tumor growth was continuously monitored using an IVIS (**Figure 7A**). Compared to the control group, both the abemaciclib and anti-PD-1 monotherapy groups exhibited delays in tumor growth, with abemaciclib achieving better tumor control than the anti-PD-1 mAb alone. However, dual treatment resulted in the most significant tumor inhibition, which was significantly greater than that achieved with either monotherapy (**Figure 7B**). Statistical results from factorial analysis also suggest that abemaciclib and anti-PD-1 worked synergistically (**Table S1**). Survival analysis showed the same trend; both single agents improved survival, while the combination of abemaciclib and the anti-PD-1 mAb led to the best survival in the ID8 model (**Figure 7C**).

To investigate whether the mice that eradicate primary tumors could develop memory response to re-challenge, we reinjected ID8 cells to the 4 mice cured by combination therapy. Another 4 naïve C57 mouse were used as controls. 2 weeks later, bioluminescence images showed that 3/4 re-challenged mice developed tumors, with 1 mouse without tumor formation. However, the tumor burden in re-challenged group was significantly lower compared to that in naïve group (**Figure 7D**).

Next, we investigated the influences of monotherapy and combination therapy on the activity and phenotype of T cells. Tumor-bearing mice were sacrificed on day 10 post treatment initiation, and TILs were extracted for flow cytometry analysis. In CD8+ T cells (**Figure 7E**), CD69, a marker for early activation, showed the highest positive rate in the combination treatment group, and this rate was significantly different from that in the other groups. In terms of the monotherapies, compared with the control group, the anti-PD-1 treatment group showed an elevated CD69-positive rate, while the abemaciclib-treated group showed only an increasing trend that was not statistically significant. CD107a and IFN-γ, markers for T cell cytotoxicity, also showed increased proportions in both the abemaciclib and anti-PD-1 mAb treatment groups; however, the combination therapy demonstrated significantly higher proportions.

Increases in the proportions of CD4+ T cells expressing CD69, CD107a or IFN-γ were also observed after combination treatment, and the positive rates were higher than those in the single-agent groups, except for CD69+CD4+ T cells, for which the percentage showed no significant difference between the anti-PD-1 treatment group and the combination treatment group, although the latter showed a higher average level (**Figure 7F**). Altogether, these results demonstrate that abemaciclib and PD-1 blockade cooperate to enhance the activation and function of both CD8+ and CD4+ T cells.

### The synergistic effect of abemaciclib and the anti-PD-1 mAb on ID8 models depends on both CD8+ T cells and B cells

**Figure 8:**
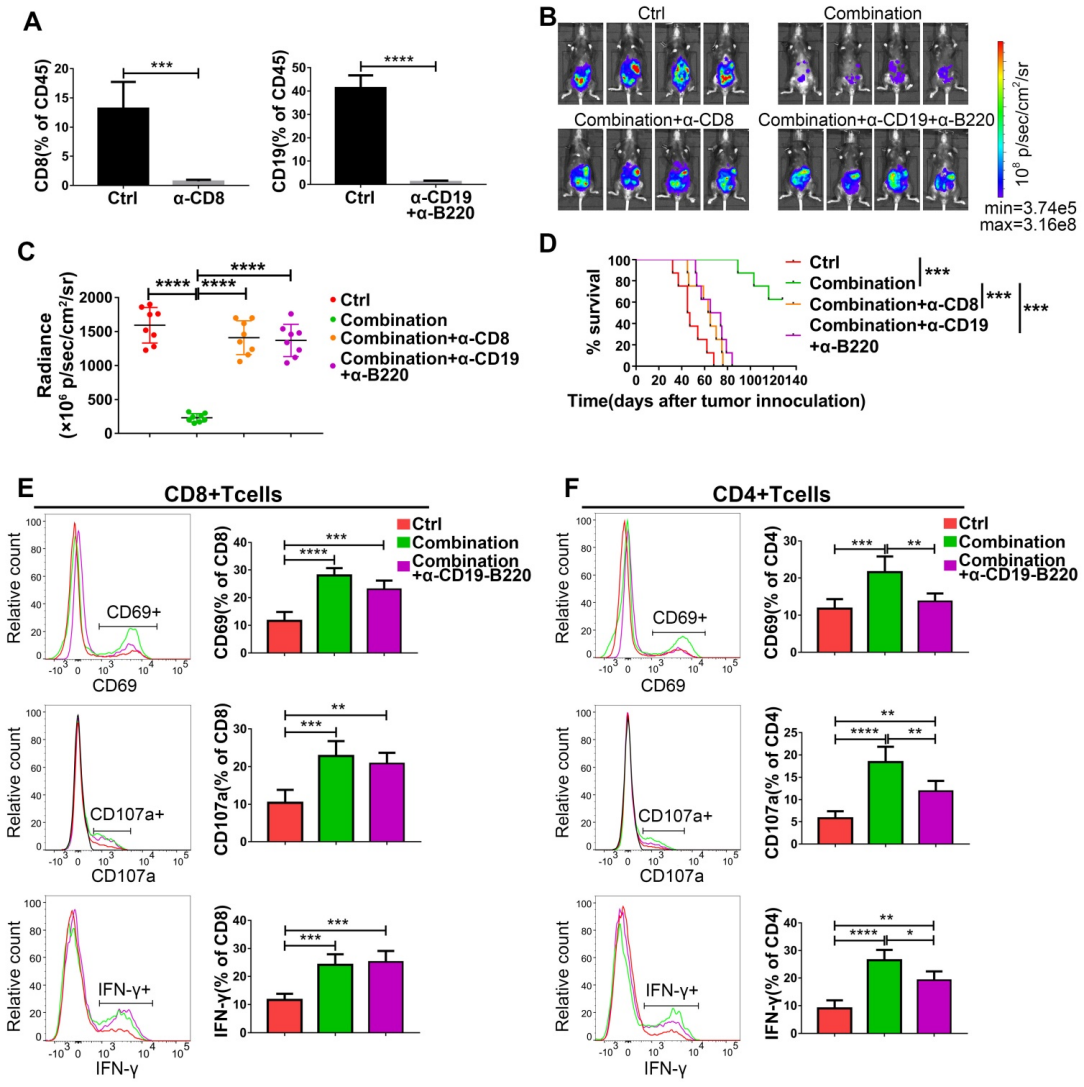
** The antitumor activity of abemaciclib plus an anti-PD-1 mAb is abrogated by CD8+ T cell depletion or B cell depletion. (A)** Flow cytometry analysis confirmed an obvious decrease in the CD8+ T cell or CD19+ B cell proportion in the mouse spleen. **(B)** Representative live animal images of ID8-luc models at day 21 post first treatment are shown. **(C)** The data were quantified with living image software, n=8 mice. **(D)** The long-term survival of ID8 tumor-bearing mice in the control, combination, combination plus CD8+ T cell depletion, and combination plus B cell depletion groups was evaluated. **(E)** The expression of CD69, IFN-γ and CD107a in the CD8+ T cell population and **(F)** CD4+ T cell populations was analyzed by flow cytometry on day 10 after treatment initiation, n=6 mice.

Based on the increased infiltration of CD8+ T cells and CD19+ B cells caused by abemaciclib treatment and the enhanced T cell activity observed in the combination treatment group, we speculate the existence of important roles for both CD8+ T cells and B cells in combination treatment. To confirm this hypothesis, we first depleted CD8+ T cells or B cells in immunocompetent C57BL/6 mice using depleting antibodies, and flow cytometry analysis showed an extremely low percentage of CD8+ or CD19+ cells in the total CD45+ spleen lymphocyte population in these mice on day 31 post tumor inoculation (**Figure 8A**). The tumor burden was heavier in CD8+ T cell- or B cell-depleted mice than in combination therapy treated-mice on day 21 post first treatment (**Figure 8B-C**). Survival analysis also showed that mice receiving the anti-CD8 depleting antibody or anti-CD19 plus anti-B220 depleting antibodies had shorter survival times than those receiving the combination treatment without depletion (**Figure 8D**).

To further investigate the possible reasons for the reduced tumor control caused by B cell depletion, we analyzed the expression of CD69, IFN-γ and CD107a on both CD8+ and CD4+ T cell populations. The expression of IFN-γ and CD107a on CD8+ T cells was not affected by B cell depletion on day 10, although a slight decrease in the frequency of CD69+CD8+ T cells was observed (nonsignificant) (**Figure 8E**). Meanwhile, the expression of all these markers was decreased in CD4+ T cells in the B cell-depleted groups (**Figure 8F**). However, both CD4+T cells and CD8+T cells exhibited decreased levels of these markers in B cell depleted group on day 15 (**Figure S1**), suggesting that the absence of B cells is associated with impaired CD4+T cell activity, which eventually led to a lack of CD8+ T cell function.

## Discussion

Ovarian cancer is the most lethal gynecological malignancy in the world, and although new treatment approaches have been approved, the improvement in survival is still limited 2. The prognostic value of TILs in ovarian cancer has been shown; thus, immunotherapy aimed at enhancing the immune response is promising. However, the efficacy of ICB in ovarian cancer has been limited 4. To improve the efficacy of immunotherapy in ovarian cancer, poor immune infiltration and the immunosuppressive TME must be overcome. In this study, we show that abemaciclib treatment can increase immune infiltration and synergize with PD-1 blockade. Dual treatment enhanced the activity of cytotoxic lymphocytes and led to improved survival in a mouse ovarian cancer model.

CDK4/6is have already been approved for the treatment of breast cancer based on their function of cell cycle control 28. However, their influence on the tumor immune microenvironment is also of concern, and preclinical studies have shown that CDK4/6 inhibition can increase antitumor immunity in lung and colon cancer 11,12. In our research, we found that treating mice with abemaciclib alone delayed tumor growth as well as ascites formation. IHC showed an increase in the number of infiltrated CD45+ lymphocytes, including CD8+ T cells, CD19+ B cells and PD-L1+ cells (**Figure 2A**). Sufficient immune infiltration is a precondition for T cell activity-unleashing therapy; however, ovarian cancer shows a low T cell-infiltrated gene expression profile, and most tumor tissues of ovarian cancer patients are poorly infiltrated 29,30. Our research found that CDK4/6i treatment promoted mixed lymphocyte recruitment and led to a proinflammatory TME in the ID8 model. From this perspective, the mixed lymphocyte infiltration induced by abemaciclib treatment is a promising strategy to convert poorly infiltrated ovarian cancer from a 'cold tumor' to a 'hot tumor'.

In accordance with this, PCR array analysis also indicated the enhanced transcription of *Cxcl10* and *Cxcl13*, the chemokine genes that play vital roles in T cell and B cell chemotaxis 26,31, while CXCL13 also plays critical roles in the formation of ELS 32. Considering the crucial role of chemotaxis in the recruitment and trafficking of immune cells into the TME, we confirmed that CDK4/6i treatment induced enhanced lymphocyte recruitment through CXCL10 and CXCL13 *in vitro*. We also found that the increased infiltration of CD3+ T cells and B cells was associated with aggregates in the form of ELS, which are ectopic lymphoid tissues formed in chronic infection site or tumors characterized by large numbers of T cells and B cells in close contact 33. In these structures, B cells are thought to play the role of antigen-presenting cells for CD8+ T cell and CD4+ T cell subsets 34. Increasing evidence indicates that such structures are correlated with prolonged survival in human cancers 33,35-38, including ovarian cancer 39,40, as they likely function as priming sites for the immune response against tumors 41.

CDK4/6i treatment increased the activity of cytotoxic lymphocytes, which is in accordance with previous reports 11,12. Immune cell phenotyping by flow cytometry showed a profound change in the TME. Although the distribution patterns of CD8+ T cells and CD4+ T cells were not significantly changed, the activities and functions of tumor-associated CD8+ T cells and CD4+foxp3- T cells were enhanced, with upregulated expression of T-bet in the CD4+foxp3- population, suggesting increased Th1 T cell development 42. PCR array analysis also indicated upregulated gene transcription of Th1 cytokines and chemokines, such as* Il2*, *Ifng* and *Cxcl10* (**Figure 5A**). In addition, CDK4/6i treatment also decreased the frequencies of M2-polarized macrophages and MDSCs, which are the main immunosuppressive cells in the TME 43-45**.**

Although CDK4/6i treatment increased immune infiltration as well as T cell activity, monotherapy did not achieve continuous tumor suppression during treatment. Considering the immunosuppressive factors present in the TME, anti-PD-1 therapy can be used to unleash the activity of cytotoxic lymphocytes. Here, we demonstrated the synergistic therapeutic effect of abemaciclib and an anti-PD-1 mAb, which may lie in the phenomenon that the increased TILs induced by abemaciclib treatment contains antitumor immune cells as well as immunosuppressive cells, such as Tregs, M2-polarized TAMs and MDSCs. The increased frequency of PD-L1+ cells observed by IHC also suggests that PD-1/PD-L1 blockade following abemaciclib may achieve a better therapeutic effect than monotherapy. Mechanistically, dual therapy promoted the activity and function of both CD8+ and CD4+ T cells to a larger extent than monotherapy, as increased expression of CD69, IFN-γ and CD107a was observed in both CD4+ and CD8+ T cells.

The antitumor role of cytotoxic T cells has long been well established, and tumor-infiltrating B lymphocytes have also been reported to correlate with patient survival. However, the role of B cells in tumor control is still controversial 46. Here, using a depleting antibody-based strategy, we demonstrated that the synergistic effect of combination treatment depended on both CD8+ T cells and B cells (**Figure 8B-D**), suggesting an antitumor role for infiltrated B cells in the ID8 model, which is in accordance with previous reports in ovarian cancer 40,47. The results from public database analysis also show that CD8 and CD19 expression are closely related to the survival of ovarian cancer patients (**Figure S2**), which suggest antitumor role of CD8+T cell and B cell. Therefore, increasing the infiltration of CD8+T cell and B cell through combination therapy is a promising strategy to improve the survival of ovarian cancer patients.

Compared to that of CD8+ T cells, the activity of CD4+ T cells is more vulnerable to B cell depletion because the expression of CD69, IFN-γ and CD107a was significantly reduced in the CD4+ population. However, this effect was minimal in CD8+ T cells. This may be partly due to the interaction between CD4+ T cells and B cells, which is crucial for enhanced activation of the local immune response and T cell cytotoxicity. CD40L, a ligand in CD4+ T cell and B cell crosstalk 48,49, showed upregulated transcription in the PCR array analysis (**Figure 5A**), suggesting enhanced T-B cell interaction upon abemaciclib treatment.

In our study, B cell depletion selectively impacts CD4+T cell activity at the early time point, however, both CD8+T cell and CD4+T cell activity was dampened at the later time point. Thus, we conclude that the effect of B cell depletion on the survival of the combination treatment group may be partly due to impaired CD4+ T cell activation and the eventual decrease in CD8+ T cell activation, which is CD4+ T cell dependent 50,51.

According to the results of a clinical trial (NCT01536743) which evaluated the efficacy and safety of palbociclib (another CDK4/6 inhibitor) in recurrent ovarian cancer, CDK4/6 inhibition with Palbociclib showed single-agent activity and is well tolerated in heavily pretreated ovarian cancer patients, with 30% of patients were free from disease progression at 6 months after treatment 52. In accordance with this, our study also find that CDK4/6 inhibition can suppress tumor progression in syngeneic mouse ovarian cancer model. However, CDK4/6 inhibitors are only approved for the treatment of breast cancer up to now. To investigate the potential of CDK4/6i in treating human ovarian cancer, exploring effective combinations to improve CDK4/6is' therapeutic efficacy are still needed. Our study demonstrated that abemaciclib induced a proinflammatory TME and thereby synergized with anti-PD-1 therapy, leading to improved survival in the ID8 model. The combination of these two agents worked synergistically to elicit cytotoxic T cell activity and achieve better tumor control than monotherapy. Meanwhile, no obvious toxicity associated with the dual treatment was observed in our study, the basic safety evaluation* in vivo* show that combination therapy had no significant effect on the body weight and serological parameters of C57 mice (**Figure S3 & Table S2**).Therefore, our study provides a rationale for the clinical use of CDK4/6is and PD-1 blockade in ovarian cancer patients.

## Supplementary Material

Supplementary figures and tables.

## Abbreviations

ICB: immune checkpoint blockade
CDK4/6: cyclin-dependent kinases 4 and 6
DMEM: Dulbecco's modified Eagle's medium
TILs: tumor-infiltrating lymphocytes
CDK4/6is: CDK4/6 inhibitors
TME: tumor microenvironment
FBS: fetal bovine serum
IVIS: In Vivo Imaging System
ELS: ectopic lymphoid-like structure
MDSC: myeloid-derived suppressor cell
TAM: tumor-associated macrophage
