# Supplementary material for: CDK4/6 inhibition promotes immune infiltration in ovarian cancer and synergizes with PD-1 blockade in a B cell-dependent manner
Source: Theranostics. 2020 Aug 25;10(23):10619–33. doi: 10.7150/thno.44871 (PMC7482823; doi:10.7150/thno.44871)
Supplement: Supplementary file 1 — Supplementary figures and tables. [file thnov10p10619s1.pdf]

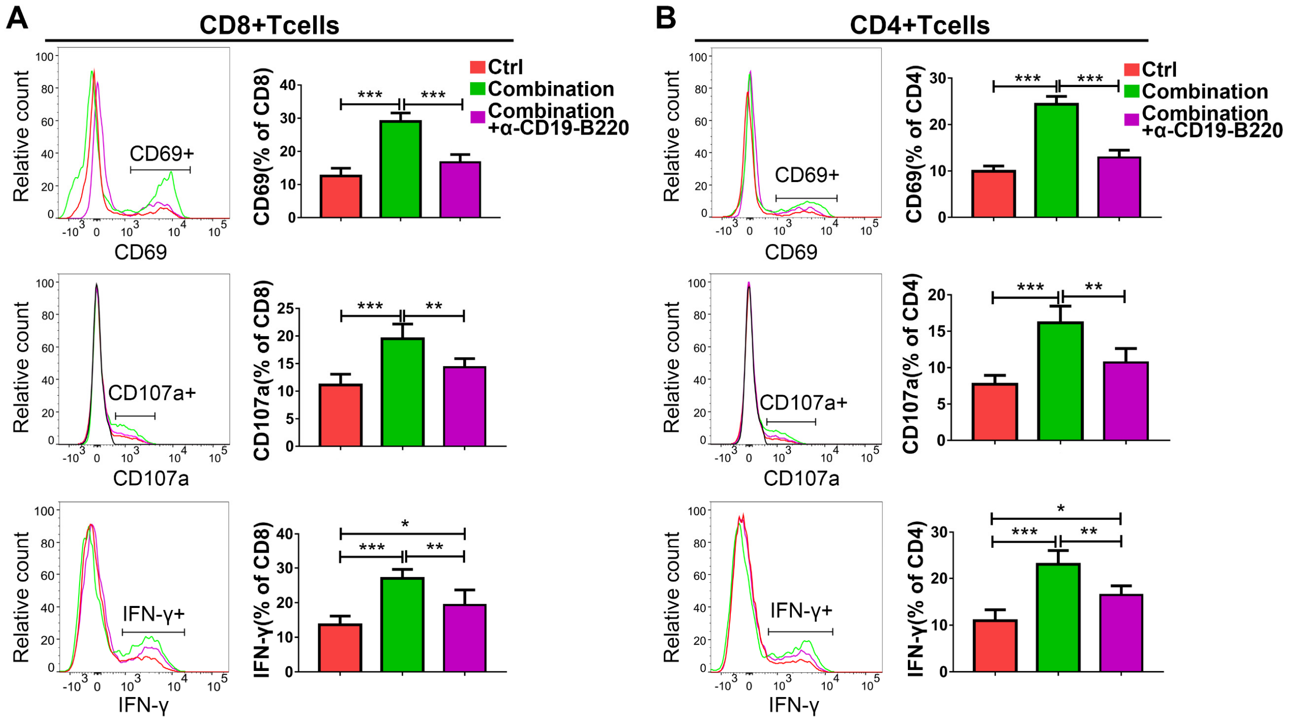

Supplementary Figure 1. B cell depletion led to impaired activation and function of both CD8+T cells and CD4+T cells. **(A)** The expression of CD69, IFN-γ and CD107a in the CD8+ T cell population and **(B)** CD4+ T cell populations was analyzed by flow cytometry on day 15 after treatment initiation, n=6 mice.

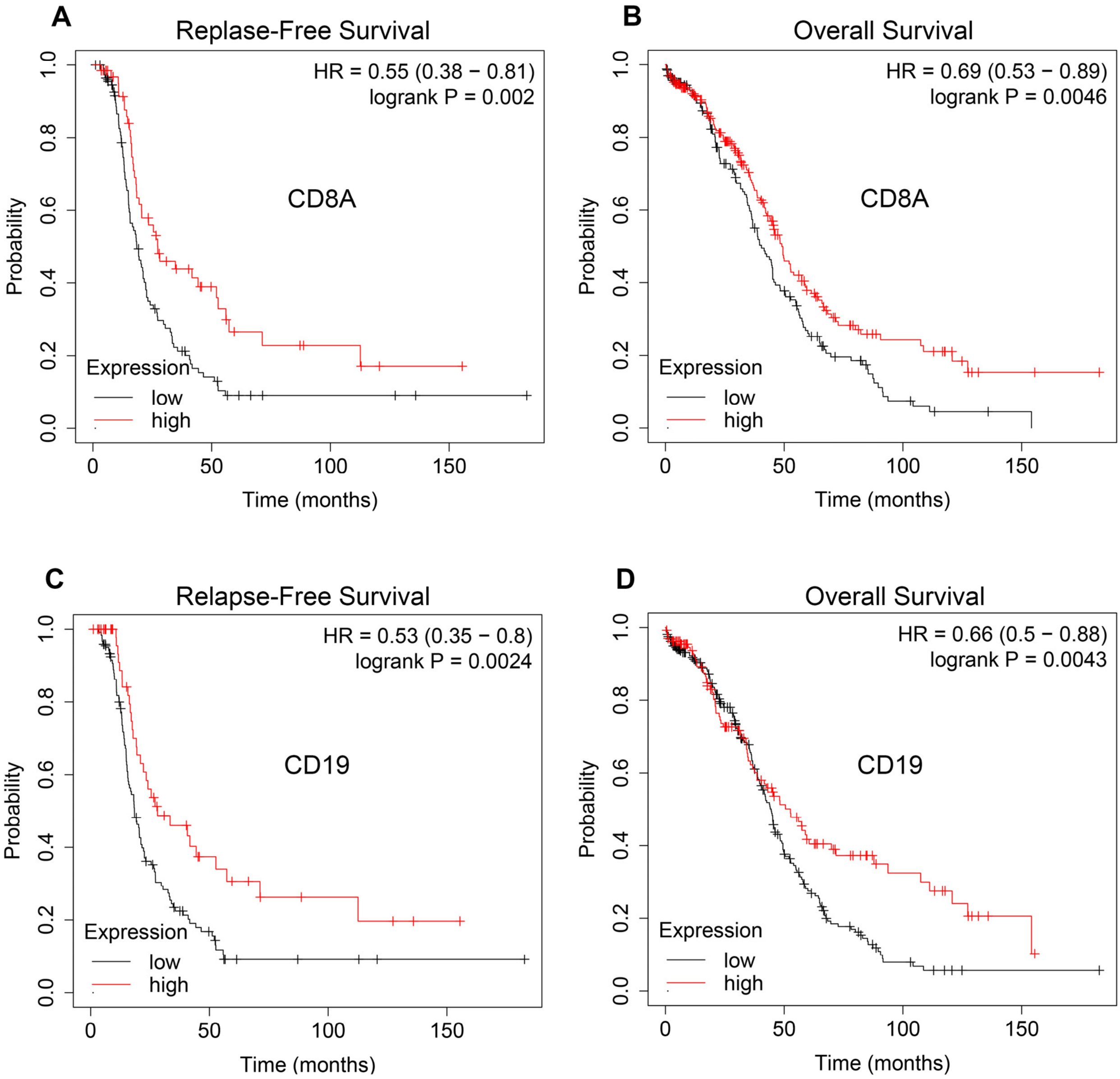

Supplementary Figure 2. Prognostic value of CD8A and CD19 expression in human ovarian cancer. Patient samples were classified as high expression (red) or low expression (black) to assess relapse-free survival (**A&C**) and overall survival (**B&D**). Data was analyzed using Kaplan Meier Plotter (www.kmplot.com).

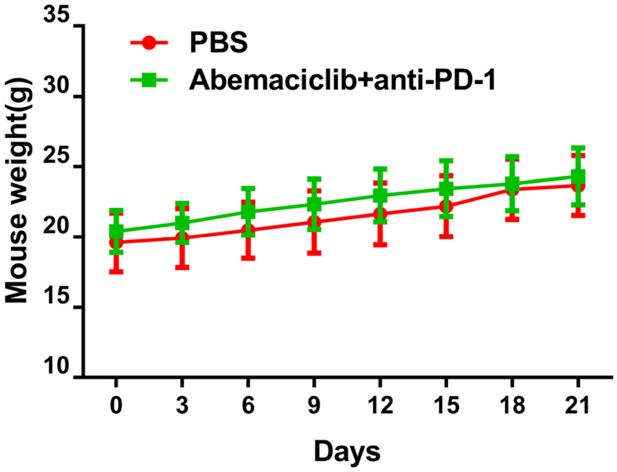

Supplementary Figure 3. Measurements of mouse weight over time. C57 mouse were administered with PBS or abemaciclib plus anti-PD-1 antibody for a totally period of 21 days, mouse weight were recorded every 3 days.

Supplementary Table1. Analysis of variance for fixed effects

	Df	Mean sq	F value	Pr＞F
**Abma**	1	6180067	98.160	5.87e-15
**α-PD-1**	1	30168	0.479	0.49109
**Time**	1	17811251	282.901	< 2e-16
**Abema: α-PD-1**	1	1429253	22.701	9.93e-06
**Abma: Time**	1	3292515	52.296	4.72e-10
**α-PD-1: Time**	1	691313	10.980	0.00146
**Abema: α-PD-1: Time**	1	524828	8.336	0.00517

Bioluminescence quantification data were analyzed in a 2×2 arrangement for factorial analysis.

Supplementary Table 2. Measurement of serological parameters after treatment

Treatment	RBC ($\times10^6$/μL)	WBC ($\times10^3$/μL)	ALT (u/L)	AST (u/L)	LDH (u/L)	BUN (mg/L)	Cr (mg/L)
**PBS**	5.9±0.2	11±1.8	24±6.7	91±13.5	683±187.2	8.1±2.6	42±0.6
**Abemaciclib +anti-PD-1**	5.4±0.3	13±3.2	25±11.2	86±16.4	659±154.1	8.7±3.2	43±0.9

Serological parameters of C57 mice were analyzed 21 days after PBS or abemaciclib +anti-PD-1 treatment. RBC: red blood cell; WBC: white blood cell; ALT: alanine aminotransferase; AST: aspartate aminotransferase; LDH: lactate dehydrogenase; BUN: blood urea nitrogen; CR: creatinine.
